# Frequency and characterization of cognitive impairments in patients diagnosed with paediatric central nervous system tumours: a systematic review

**DOI:** 10.3389/fonc.2023.1198521

**Published:** 2023-05-19

**Authors:** Francesco Sciancalepore, Francesco Fabozzi, Giulia Albino, Giada Del Baldo, Valentina Di Ruscio, Beatrice Laus, Danilo Menegatti, Roberto Premuselli, Domitilla Elena Secco, Alberto Eugenio Tozzi, Eleonora Lacorte, Nicola Vanacore, Andrea Carai, Angela Mastronuzzi, Allena-Mente Study Group

**Affiliations:** ^1^ National Center for Disease Prevention and Health Promotion, Italian National Institute of Health, Rome, Italy; ^2^ Department of Oncology/Hematology, Cell Therapy Gene Therapies and Hemopoietic Transplant, Scientific Institute for Research, Hospitalization and Healthcare Bambino Gesù Children’s Hospital, Rome, Italy; ^3^ Department of Pediatrics, University of Tor Vergata, Rome, Italy; ^4^ Department of Experimental Medicine, Sapienza University of Rome, Rome, Italy; ^5^ Hispalense Institute of Pediatrics, Seville, Spain; ^6^ University of Rome “La Sapienza”, Department of Computer, Control, and Management Engineering (DIAG), Rome, Italy; ^7^ Multifactorial and Complex Diseases Research Area, Scientific Institute for Research, Hospitalization and Healthcare Bambino Gesù Children’s Hospital, Rome, Italy; ^8^ Neurosurgery Unit, Scientific Institute for Research, Hospitalization and Healthcare Bambino Gesù Children’s Hospital, Rome, Italy

**Keywords:** brain tumours, cognitive deficits, paediatric oncology, neuropsychology, central nervous system

## Abstract

**Background:**

This systematic review has been conducted with the aim of characterizing cognitive deficits and analyzing their frequency in survivors of paediatric Central Nervous System tumours.

**Materials and methods:**

All literature published up to January 2023 was retrieved searching the databases “PubMed”, “Cochrane”, “APA PsycInfo” and “CINAHL”. The following set of pre-defined inclusion criteria were then individually applied to the selected articles in their full-text version: i) Retrospective/prospective longitudinal observational studies including only patients diagnosed with primary cerebral tumours at ≤ 21 years (range 0-21); ii) Studies including patients evaluated for neuro-cognitive and neuro-psychological deficits from their diagnosis and/or from anti-tumoral therapies; iii) Studies reporting standardized tests evaluating patients’ neuro-cognitive and neuro-psychological performances; iv) Patients with follow-ups ≥ 2 years from the end of their anti-tumoral therapies; v) Studies reporting frequencies of cognitive deficits.

**Results:**

39 studies were included in the analysis. Of these, 35 assessed intellectual functioning, 30 examined memory domains, 24 assessed executive functions, 22 assessed attention, 16 examined visuo-spatial skills, and 15 explored language. A total of 34 studies assessed more than one cognitive function, only 5 studies limited their analysis on a single cognitive domain. Attention impairments were the most recurrent in this population, with a mean frequency of 52.3% after a median period post-treatment of 11.5 years. The other cognitive functions investigated in the studies showed a similar frequency of impairments, with executive functions, language, visuospatial skills and memory deficits occurring in about 40% of survivors after a similar post-treatment period. Longitudinal studies included in the systematic review showed a frequent decline over time of intellectual functioning.

**Conclusions:**

Survivors of paediatric Central Nervous System tumours experience cognitive sequelae characterized by significant impairments in the attention domain (52.3%), but also in the other cognitive functions. Future studies in this research field need to implement more cognitive interventions and effective, but less neurotoxic, tumour therapies to preserve or improve neurocognitive functioning and quality of life of this population.

## 1 Introduction

Central nervous system (CNS) tumours represent the most common paediatric solid neoplasm in childhood, accounting for approximately 25% of childhood cancers (1, 2). Although CNS tumours are currently the leading cause of death from cancer in children less than 20 years of age, the overall survival (OS) is now approximately 75% (2, 3). Given the increase in the cure rate, more attention must be paid to treatment-related late effects among childhood cancer survivors. In particular, survivors of CNS tumours experience often devastating neurocognitive sequelae involving several domains such as attention, processing speed, executive function, memory, and intelligence (4–6). These deficits affect interpersonal relationships, emotional functioning, employability and independent living, resulting in an impaired quality of life (QOL) (6). Accordingly, survivors of CNS tumours might also exhibit decline in academic performances over time, particularly in reading, spelling, and arithmetic (7).

Evidence suggests neurocognitive outcomes can be affected by both treatments and the tumour itself. Neurosurgery has been found to yield cognitive impairments of lesser severity than chemotherapy or radiation therapy (8). Surgical sequelae depend primarily on tumour location, while radiotherapy is the most recognized risk factor for neurocognitive dysfunctions, causing white matter injury as well as vasculopathies (5). Chemotherapy effects have also been found to be associated with poor intellectual function (6). At the same time, clinical presentation at diagnosis can impact outcomes, with hydrocephalus and seizures associated with worse cognitive function (9, 10). In addition, younger age at diagnosis and treatment represents an increased risk for late sequelae, since the developing brain is more vulnerable to both disease and treatment-related injury (11).

However, despite the growing interest regarding this topic and the considerable amount of studies and qualitative reviews published, in recent years only a few systematic reviews and meta-analysis have been published providing an overview of the most common cognitive impairments in survivors of paediatric CNS tumours (12, 13). Moreover, no systematic reviews have been conducted with the aim of quantifying the frequency of neurocognitive sequelae in paediatric CNS tumours survivors.

## 2 Material and methods

This systematic literature review was performed according to the methodology described in the Cochrane handbook for systematic reviews (14) and was reported based on the PRISMA statement for reporting systematic reviews and meta-analyses (15). All literature published up to January 2023 was retrieved searching the databases “PubMed”, “Cochrane”, “APA PsycInfo” and “CINAHL” using the following search query: (cognit* OR neuropsyc* OR neuro-psyc* OR memor* OR attention* OR “problem solving” OR problem-solving OR executive OR “visual motor” OR visual-motor OR verbal* OR language*) AND (impairment* OR deficit* OR assessment* OR disorder*) AND (child* OR infant* OR adolescent* OR pediatri* OR paediatr* OR poediatr*) AND (cancer* OR tumor* OR neopla* OR malignan* OR *irradiation* OR *proton* OR chemother* OR radiother* OR radio-ther* OR chemo-ther*) AND (cereb* OR brain* OR *crani* OR cns OR spin* OR medull*).

No limitations in the search strategy were applied to the date of publication, study design, or language. References of considered studies were also searched to identify any further relevant data.

The records identified by the search were uploaded on “Rayyan” (16), a website for systematic reviews, to organize the study selection in a more efficient way. The titles and abstracts of the identified records were initially screened and selected by seven groups composed of two independent and blinded reviewers (P.P.+A.M., P.P.+G.D.B., M.G.+F.F., M.G.+D.E.S., V.M.C+V.D.R., V.M.C+G.A., F.S.+B.L.) based on their pertinence and relevance to the review topic. Conflicts and disagreements were resolved by consensus.

The following set of pre-defined inclusion criteria were then individually applied to the selected articles in their full-text version: i) Retrospective/prospective longitudinal observational studies including only patients diagnosed with primary cerebral tumours at ≤ 21 years (range 0-21); ii) Studies including patients evaluated for neuro-cognitive and neuro-psychological deficits from their diagnosis and/or from anti-tumoral therapies; iii) Studies reporting standardized tests evaluating patients’ neuro-cognitive and neuro-psychological performances; iv) Patients with follow-ups ≥ 2 years from the end of their anti-tumoral therapies; v) Studies reporting frequencies of cognitive deficits.

Case reports, case series, reviews, letters, conference proceedings, abstracts and editorials were excluded. Articles not published in English were removed. Systematic reviews were considered separately to check the consistency of the data. Data extraction from the included studies was performed by all the reviewers involved in the previous phases by using standardized tables.

## 3 Results

Bibliographic searches on literature databases yielded 6,979 records. After a first screening, 647 records were selected, and the related full texts were retrieved. Then, 608 full texts were further excluded, as they did not meet the inclusion criteria. Overall, 39 studies were finally included. Of these, 30 studies were cross-sectional as they assessed cognitive functions only once post-treatment and 9 had a longitudinal design since they evaluated cognitive outcomes at least twice during the study period (pre and post-treatment).

**Figure 1:**
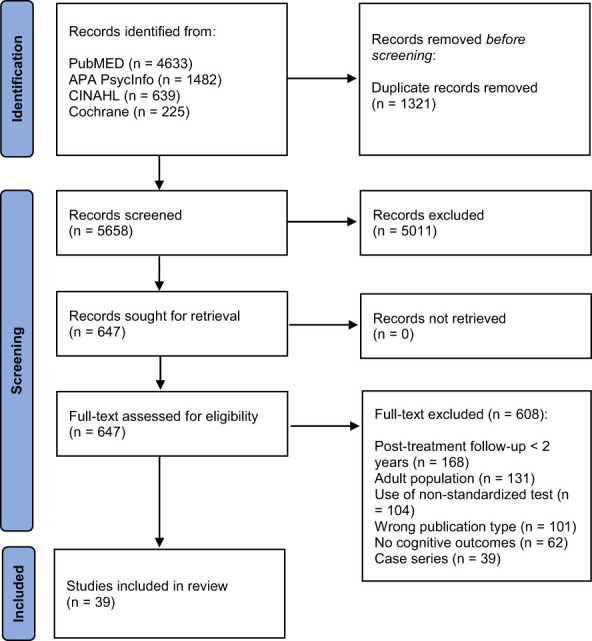
PRISMA Flow-chart of included studies.

A high agreement (>95%) was reported by the reviewers involved in the study selection process and conflicts in the screening process were resolved by interpersonal discussion. The flow diagram of included studies is reported in **Figure 1**.

### 3.1 Demographic and clinical characteristics of the population

All the 39 studies enrolled patients with a diagnosis of CNS tumour before 21 years of age. The samples across the studies ranged from a number of 10 to 120 patients enrolled (total: 1613, mean: 41.4), a higher number of studies (63.2%) enrolled more males than females. The mean age at diagnosis and at the cognitive assessment ranged from 29 months to 11.6 years and from 35.9 months to 32.6 years, respectively. The principal tumour types included were: medulloblastomas (reported in 24 studies), ependymomas (reported in 14 studies), astrocytomas (reported in 13 studies), and gliomas (reported in 11 studies). Patients were commonly treated with surgery (10 studies), chemotherapy (6 studies), radiation therapy (9 studies), or combined cancer therapy (29 studies). In all the studies, patients were examined after a minimum of 2 years from the end of the therapies. In longitudinal studies, the mean follow-up duration of the neuropsychological assessments ranged from 24 months to 12 years.

Additional information is reported in **Supplementary Table 1**.

### 3.2 Quality assessment of the studies

The quality of the 39 studies reporting data about the frequency of cognitive deficits was assessed through the Methodological Evaluation of Observational REsearch (MORE) checklist (17). In this checklist higher scores are associated to worse study quality, since for each item a score of 0 is assigned if there is no flaw, a score of 1 if there is a minor flaw and a score of 2 if there is a major flaw. The missing item (Not Reported/Not Applicable) are not considered in the count, but their characterization is important to define the study quality. Studies with a same total score might present a different quality due to the different number of Not Reported or Not Applicable items.

The quality of these studies ranged from low to high, with most of the studies (64.2%) showing a medium quality (medium overall score: 6.4, SD 2.7). None of the included studies obtained 0 points. The highest score achieved was 1 (18–20) and the lowest score was 13 (21), with many studies not reporting several items concerning the external validity.

The main reasons associated with a poorer quality were related to items concerning the external validity: a low response rate of the sample, the absence of the exclusion rate from the analysis and flaws regarding the sampling of the subjects. On the other hand, most of the studies showed positive results related to the internal validity, with only minor flaws in defining, reporting and measuring the cognitive outcomes. Moreover, most of the included studies did not report any confidence intervals or other measures of precision of the estimates. A summary of the quality assessment of the included studies is shown in **Table 1S** and **Table 2S**.

### 3.3 Intellectual functioning

**Figure 2:**
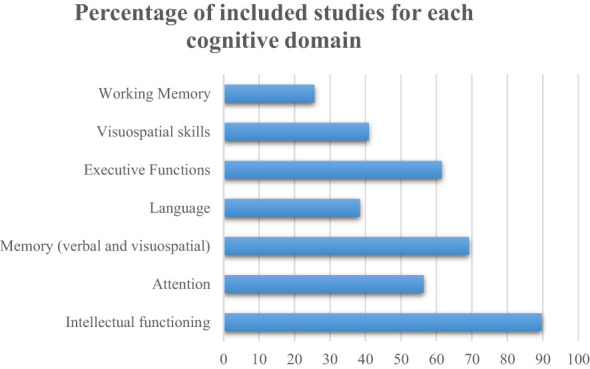
Percentage of included studies for each cognitive domain.

**Figure 3:**
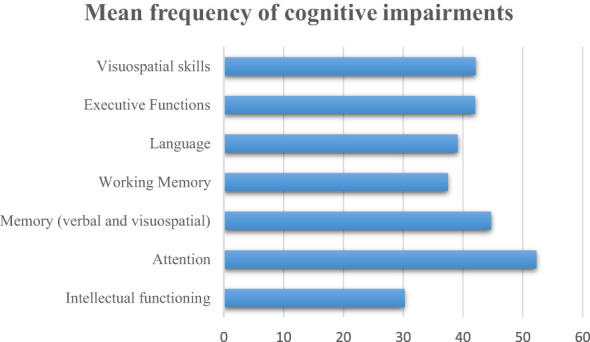
Mean frequency of cognitive impairments.

Almost all the studies (89.7%) assessed intellectual functioning (**Figure 2**). The most employed scale to assess this cognitive function was the Wechsler Scale, specifically the Wechsler Intelligence Scale for Children (WISC), the Wechsler Preschool and Primary Scale for Intelligence (WPPSI) to assess paediatric patients, and the Wechsler Adult Intelligence Scale (WAIS) to assess adult survivors. Most of the studies (74.2%) assessed intellectual functioning through a cross-sectional design, examining the cognitive domain only once during the study period. These studies reported a mean frequency of intellectual deficits of 30.3% (range: 0-75) after a mean period that ranged from 2.5 to 20.7 post-treatment years (**Figure 3**).

**Figure 4:**
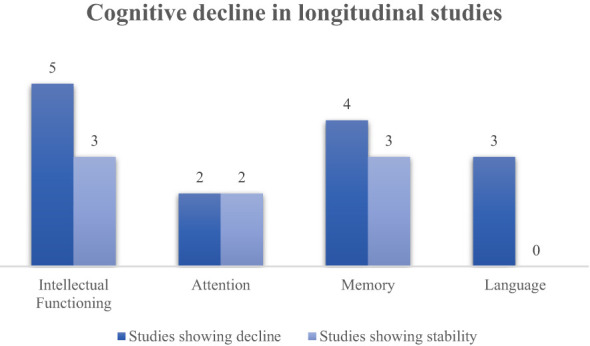
Cognitive decline in longitudinal studies.

Nine studies (22–30) evaluated the trajectory of intellectual functioning by assessing the cognitive function at least twice during the study period; the mean follow-up duration of the studies was 4.6 years (range: 2-12). Most of these longitudinal studies showed an increasing impairment of IQ during the follow-up (**Figure 4**).

Hoppe-Hirsch et al. (24) showed a significant decline of IQ: five years after the treatments, 42% of patients with medulloblastoma previously treated with surgery and radiation therapy exhibited an IQ < 80 (18% of patients an IQ < 60); at 10-year evaluation 84% of the patients developed an IQ < 80 (46% patients an IQ < 60) and only 15% maintained an IQ above 80. In addition, the study by Packer et al. (25) documented a decline of general IQ over 2 years in patients with CNS tumours receiving whole-brain radiotherapy and chemotherapy: the mean full-scale IQ (FSIQ) for children was 105 at baseline, 97 at Year 1, and 91 at Year 2. Another recent study (28) documented a decline of intellectual functioning over 5 years in patients with heterogeneous CNS tumours treated with surgery and radiation therapy: the frequency of IQ impairments increased from 60% before surgery to 90% 5 years-post-treatment.

Additionally, Palmer and colleagues (26) examined patterns of intellectual development among 44 survivors of paediatric medulloblastoma, showing an increasing decline in IQ values during 12 years of neuropsychological assessments. The first assessment (1 year after the treatment) showed that 18% of the patients exhibited an IQ below the expected population norm, while at the last evaluation (12 years after the treatment) 83.3% of the survivors had developed intellectual deficits: the obtained mean estimated FSIQ of 83.57 was more than one standard deviation (SD) below the expected population norm. The study by Fay et al. (22) also indicated a decline in FSIQ over the years, although the mean FSIQ values remained in the broadly average range at both time points.

Even though the studies mentioned above indicated a general decline over time of intellectual functioning, some studies showed a different trend. For instance, the study by Youn (30) reported that a stable global IQ was observed throughout the observation period in patients with different CNS tumours; another research study (29) showed that 4.5 years after the completion of surgery and radiation therapy only 8.7% of the patients diagnosed with ependymoma exhibited deficits in the intellectual functioning; the study by Sands et colleagues (27) examined intellectual skills over a mean period of 2.75 years in a heterogeneous population with different CNS tumours and authors documented that IQ scores remained stable over the follow-up period, although a moderate percentage of impaired patients (33.3%).

### 3.4 Attention

A total of 22 studies (56.4%) assessed attention. The most employed scales were the Continuous Performance Test (CPT/CPT-II), the Cancellation Test, and the Trail Making Test A and B (TMT-A, TMT-B). Eighteen of these studies (81.8%) evaluated attention by assessing patients only once during the study period; these studies reported a mean frequency of attention impairments equal to 52.3% (range: 16-85) after a mean period that ranged from 2.5 to 22.9 years-post-treatment.

Four studies (22, 23, 28, 30) examined the trajectory of attention domain in survivors of CNS tumours, by assessing the population within a mean follow-up of 3.9 years (range: 2.5-5) between the first and the last neuropsychological assessment.

A decline of attention was shown in the study by Fay et al. (22) that reported a cognitive decline over time (2.4 years) in attention and processing speed in patients diagnosed with medulloblastoma and previously treated with chemotherapy or radiotherapy. Furthermore, the study by Söderström (28) reported a decline of attention between the pre-surgery period and after the end of radiation therapy.

On the other hand, Heitzer and colleagues (23) showed that 32% of low-grade glioma survivors, exclusively treated with surgery, exhibited attention impairments at the first neuropsychological assessment. However, the authors highlighted a stability of attention impairments from baseline to the last neuropsychological evaluation (up to 6 years). Another study (30) documented a stable pattern of attentional impairments over a 5 year-period.

### 3.5 Memory

Memory impairments were documented in several studies (76.9%). The most employed tests to assess verbal memory were the Rey Auditory-Verbal Learning Test (RAVLT), the California Verbal Learning Test (CVLT) and the Digit Span forward, while visuospatial memory was often assessed with the immediate and delayed recall of the Rey Complex Figure Test (RCFT). Moreover, verbal and visuospatial memory were evaluated through the Wechsler Memory Scale and subtests from neuropsychological batteries like the Kaufman Assessment Battery for Children-II (KABC-II) and NEPSY-II. The working memory domain was assessed through the Digit Span Backwards or subtests from the Wechsler Memory Scale.

A total of 23 out of 30 studies (76.7%) assessed memory functions through a cross-sectional design, examining the cognitive domain only once during the study period. Specifically, the studies reported a mean frequency equal to 33.1% (range: 8.7-54) of verbal memory impairments after a mean period post-treatment that ranged from 2.8 to 22.9 years; 56.3% (range: 25-80) of visuospatial memory impairments after a mean period post-treatment that ranged from 3 to 22.9; 37.5% (range: 20-73) of working memory dysfunctions after a mean period post-treatment that ranged from 2.5 to 22.9 years.

Seven studies (22, 23, 25, 27–30) investigated the longitudinal course of memory outcomes in survivors of CNS tumours, by assessing the patients within a mean follow-up of 3.5 years (range: 2-5) between the first and the last neuropsychological assessment.

The study by Fay et al. (22) reported that 47% and 36% of patients with medulloblastoma were impaired in verbal and visual memory, respectively, after a mean of 3.5 years from diagnosis. In addition, working memory was compromised in 14% of the children, and the authors documented a further decline of this cognitive function over a period of 2.5 years between the two neuropsychological assessments. A decline was also found in another study (28) that reported a significant decline in memory functioning after 5 years from the first baseline assessment, specifically in the Working Memory Index (WMI), in survivors of heterogeneous CNS tumours treated with surgery and radiotherapy. Additionally, Packer and colleagues (25) revealed a memory decline over a period of 2 years in children receiving whole-brain radiotherapy and chemotherapy. Significant memory impairment was evident for only a few children at baseline but at 2 years almost half (42%) performed below the normal range in list learning, while 64% had significant difficulty with immediate auditory recall. One study (29) assessed patients diagnosed with ependymoma treated with combined cancer therapy, revealing that after a median follow-up of 4.5 years, short-term memory was significantly diminished in 1 of 10 (10%) patients and long-term memory measured by word list was significantly diminished in 3 of 19 (15.8%) tested patients.

On the other hand, the studies by Heitzer (23), Sands (27) and Youn (30) documented a stable frequency of memory impairments over time (follow-ups of 3, 2 and 5 years, respectively), even if the population and the treatment modalities of the studies were heterogeneous.

### 3.6 Language

Fifteen studies (38.5%) assessed language domains. The most employed scales were the Peabody Picture Vocabulary, the Boston Naming test and vocabulary subtests from the Wechsler Scales.

Twelve of these studies evaluated language by assessing patients only once during the study period, reporting a mean frequency of language impairments equal to 39.1% (range: 0-100) after a period that ranged from a mean of 2.9 to 22.9 years-post-treatment.

Three studies (22, 25, 27) examined the trajectory of the language domain by assessing the population within a mean follow-up of 2.4 years (range: 2-2.8) between the first and the last neuropsychological assessment.

Packer et al. (25) documented a 2-year-decline of language in a population with malignant CNS tumours. The neuropsychological assessments were performed before and after the therapies, showing an increase in impaired patients over time, from a frequency of 18.1% to 50%. According to this study, Sands and colleagues (27) found a decline in a heterogeneous population with CNS tumours: the authors documented a frequency of language dysfunctions equal to 43.8% after a mean follow-up of 2.75 years, with most of the patients that exhibited a score in the low-average range.

Another study (22) that enrolled children diagnosed with medulloblastoma and treated with irradiation therapy found a frequency of 29% of impaired patients after a neuropsychological follow-up of 2.5 years.

### 3.7 Executive functions

A total of 24 studies (61.5%) assessed Executive Functions in patients diagnosed with childhood CNS tumours. The most employed scales to assess this cognitive function were the Wisconsin Card Sorting Test (WCST), the Modified Card Sorting Test (MCST), the Stroop Test, and tests assessing Verbal Fluency. Most of these studies (79.2%) assessed Executive Functions through a cross-sectional design, examining the cognitive domain only once during the study period. The studies reported a mean frequency of executive impairments equal to 42% (range: 13-73) after a post-treatment period ranging from a mean of 3.4 to 22.9 years.

Five studies (22, 23, 28–30) evaluated the trajectory of executive functions by assessing this cognitive domain at least twice during the study period; the mean follow-up duration of these studies was 4 years (range: 2.5-5). However, only one study (29) provided a frequency of executive impairments at the latest neuropsychological assessment. In this study, 12 patients diagnosed with ependymoma received the WCST and results revealed that 7/12 (58.3%) showed deficits with slow adaption, tendency to keep one strategy, difficulties with reasoning, and problems to maintain the intentional thread after a follow-up of 4.5 years.

### 3.8 Visuospatial skills

Visuospatial skills were evaluated in 16 studies (41%). The most employed scales to assess this cognitive function were the Copy version of the Rey Complex Figure Test (RCFT), the Judgment of Line Orientation Test (JLOT), and the Visual-Motor Integration Test (VMI).

Twelve studies (75%) assessed visuospatial skills by monitoring patients only once during the study period, reporting a mean frequency of visuospatial impairments of 42.1% (range: 6.3-73.5) after a post-treatment period ranging from a mean of 3.4 to 22.9 years.

Four studies (22, 25, 28, 29) examined the trajectory of visuospatial domain in survivors of CNS tumours by assessing the population within a mean follow-up of 3.5 years (range: 2-5) between the first and the last neuropsychological assessment. Only two of these studies provided a frequency of visuospatial impairments at the latest neuropsychological assessment. Packer and colleagues (25) reported a stable frequency of visuomotor and visuospatial dysfunctions in children diagnosed with heterogeneous tumours. The authors found visuospatial impairments at initial testing in 4 patients (36.4%) and at 2-years follow-up testing in five (35.7%). Concerning visuospatial capacities, another study (29) found heterogeneous outcomes. Authors documented that after 4.5 years from the first baseline assessment, 3 of 16 (18.8%) survivors of ependymoma had severe difficulties in reproducing the RCFT, while none of the 11 patients tested had severe difficulties with the Benton line orientation test.

## 4 Discussion

In this systematic review we summarized the available evidence about the frequency of cognitive impairments occurring in patients diagnosed with childhood primary CNS tumours by focusing also on the characterization of these dysfunctions. The studies we have included in this paper enrolled survivors of paediatric CNS tumours who had completed oncological treatments at least 2 years earlier.

We included 39 studies which examined different cognitive functions. Of these, 35 assessed intellectual functioning, 30 examined memory domains, 24 assessed executive functions, 22 assessed attention, 16 examined visuo-spatial skills, and 15 explored language. A total of 34 studies assessed more than one cognitive function, only 5 studies (28, 31–34) limited their analysis on a single cognitive domain.

Overall, evidence from this systematic review suggests that attention impairments are the most recurrent in this population, with a mean frequency of 52.3% after a median period post-treatment of 11.5 years. These results are consistent with other studies reporting attentional deficits as one of the most common impairments in cancer survivors (35, 36). Two longitudinal studies (22, 28) have also documented an increased frequency of this impairment, indicating a significant decline of this function over time. However, other two studies (23, 30) depicted a different scenario characterized by a stable pattern of attentional deficits during the follow-up.

The other cognitive functions investigated in the studies showed a similar frequency of impairments, with executive functions, language, visuospatial skills and general memory deficits occurring in about 40% of survivors after a similar post-treatment period. However, memory function showed different trends depending on the type of memory domain examined in the neuropsychological assessments. Specifically, visuospatial memory resulted the most compromised function, affecting a mean of 56.3% survivors. On the other hand, verbal memory and working memory impairments occurred in 33.1% and 37.5% of patients, respectively.

Intellectual functioning was the most explored cognitive domain, yet, at the same time, had a lower frequency of impairment (30.3%) among survivors of CNS tumours. The majority of longitudinal studies included in this work (22, 24–26, 28) reported an important decline of IQ during the follow-up. Accordingly, in literature other studies also documented a decline of this cognitive function over time (37, 38), indicating this function as one of the most important cognitive domains to be monitored in cancer survivors. On the other hand, evidence also suggest a different scenario with IQ values characterized by a stability or, in some cases, by an increase over the years (30, 39).

To resolve and understand these contrasting results, there is the need to highlight the extreme heterogeneity of the studies published in literature and those we have examined in this systematic review. To this regard, in these studies we observed four main types of heterogeneity: clinical, treatment-related, demographic (age) and related to the study design. The first variables to be addressed are certainly represented by the clinical and treatment heterogeneity of the studies. Specifically, evidence showed that some CNS tumours and relative treatments are associated to worse neuropsychological sequelae when compared to other ones. Some cross-sectional comparative studies have highlighted less severe deficits in IQ in survivors of ependymoma treated with irradiation confined to the posterior fossa alone, or in patients treated for low-grade astrocytoma by use of surgery alone, than in those treated for medulloblastoma with craniospinal radiotherapy (4). Accordingly, results emerging from eight studies included in this systematic review (24, 26, 32, 40–44) showed that a mean of 45% of patients with a diagnosis of childhood medulloblastoma exhibited intellectual impairments at the latest assessment, compared to a mean of 25.8% in survivors of other CNS tumours. Moreover, evidence from included studies reported a possible impact on IQ dysfunctions according to the type of treatment employed: a mean frequency in IQ deficits of 14.4% was observed in patients exclusively treated with surgery, a mean of 33.4% in patients treated with chemotherapy and radiotherapy and a mean of 39.1% of those who had previously been treated with surgery and chemo-irradiation therapy. Also, other cognitive functions are affected by the type of clinical diagnosis and treatment received during the paediatric age. A study (45) analysed a group of patients treated for medulloblastoma and another group treated for astrocytoma. The medulloblastoma group was treated with surgery and radiotherapy, while patients with astrocytoma underwent only surgery. The first group performed poorer than the astrocytoma group on all neuropsychological measures (attention, psychomotor speed and visual memory) except one (motor speed in dominant hand). Youn et al. (30) observed a different trajectory of cognitive impairments according to the type of tumour and treatment. Patients with posterior fossa tumours exhibited a wide range of neuropsychological impairments (attention, executive functions, and motor dysfunctions) reflecting the effect of cerebellar dysfunctions. However, these impairments did not worsen over time after proton therapy. In contrast to posterior fossa tumour group, the group diagnosed with germ cell tumours demonstrated a relatively preserved global IQ, but their processing speed and memory function were significantly impaired at baseline with memory dysfunctions declining over time. Furthermore, from the studies included in this review a mean of 28.5% of patients treated only with surgery (children often diagnosed with low-grade gliomas or astrocytomas) displayed at least one cognitive impairment at the latest assessment, while a mean of 40.9% of patients previously treated with chemotherapy and/or radiation therapy and a mean of 39.9% of patients who underwent surgery and chemo-irradiation therapy exhibited cognitive impairments at the latest assessment. These results are in line with studies published in literature suggesting that a higher impact of radiation therapy on cognitive impairments is due to aberrations of white matter development (4). In patients treated with surgery alone, these pathways seem to be relatively preserved given the focal nature of the tumour and treatment (46).

**Figure 5:**
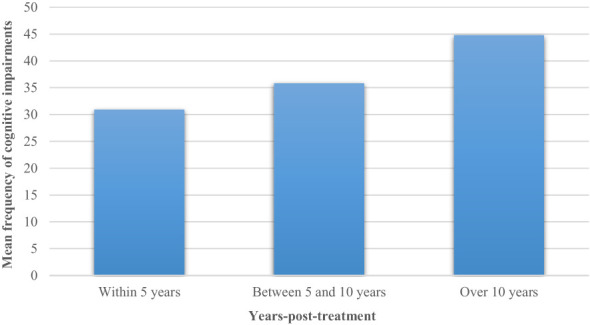
Mean frequency of cognitive impairments stratified for post-treatment years.

Another variable to be addressed to examine the heterogeneous outcomes emerging from this work is represented by the age at diagnosis/treatment and at assessment. Specifically, a different frequency of cognitive impairments might be associated to the timing of neuropsychological assessment. A meta-analysis (12) found that longer time since diagnosis was highly associated with worse intellectual outcomes in survivors of CNS tumours. Our systematic review confirmed this finding: as depicted in **Figure 5** we found a growing frequency of impaired patients as the years since the end of treatments increased (30.9% within 5 years, 35.8% between 5 and 10 years, 44.8% over 10 years). These results might be explained by the patients’ age at the start of oncological treatment. Indeed, it has been suggested that age at radiation therapy is a proxy variable for underlying neurodevelopmental maturity (47). Although development of cortical grey matter peaks at approximately 4 years of age, cortical white matter volume increases steadily until 20 years of age (48). Therefore, patients who are younger when they receive radiation therapy generally have less fully developed white matter. However, because both younger and older patients have been demonstrated to lose white matter volume at a similar rate (49), the younger irradiated patients continue to display reduced total white matter volume years after treatment. These deficits in white matter volume among younger patients have been associated with a growing cognitive morbidity (26). Therefore, time since treatment is an important determinant of neurocognitive deficits, as the deficits often increase over time, owing to a slower rate of acquiring new skills and knowledge compared with healthy peers (12). To this regard, in this review most of the studies were cross-sectional and did not allow to outline the cognitive evolution of the patients during a follow-up; conversely, longitudinal studies have allowed a more accurate analysis of the cognitive trajectory of CNS survivors. However, longitudinal studies represented only the 23.1% of the included studies, thus more longitudinal studies are needed in future to address how the frequency of cognitive impairments change over time in this population.

Despite some limitations and heterogeneities across all the studies, results from this systematic review depict a scenario characterized by significant late cognitive sequelae in these survivors. Attentional deficits are the most recurrent in this population, but also other functions are affected in a relevant way. These impairments are often influenced by several factors such as clinical diagnosis, treatment type, and years from diagnosis or treatment. Since several longitudinal studies (22, 24, 28) showed a neurocognitive decline over time, survivors of CNS tumours need to be monitored and trained to preserve or improve their cognitive functions. To this regard, a recent systematic review (50) described the importance of cognitive-based computer interventions in improving cognitive functions of paediatric patients with brain tumours. Nevertheless, some of these studies revealed only transient positive effects on these neurocognitive domains with a significant number of dropouts during the follow-up. Moreover, the interventions were often limited to train working memory, attention and executive functions. There is a need to develop more cognitive interventions that are also focused on other impaired domains such as visuospatial skills, language and memory (visuospatial memory and verbal memory). There is also an imperative need for effective, but less neurotoxic, tumour therapies. Until such therapy is developed, cognitive, behavioural, pharmacological, and environmental interventions will be needed to address the neurocognitive effects that cannot be avoided. These precautions might be fundamental to enhance academic achievement and quality of life of survivors diagnosed with paediatric CNS tumours.

## 5 Conclusions

This work aimed to systematically review the evidence about the frequency and characterization of cognitive deficits occurring in survivors of paediatric CNS tumours. Studies showed that attentional impairments were the most widespread in this population with a mean frequency of 52.3% after a median period post-treatment of 11.5 years. Other cognitive domains, such as language, memory, visuospatial skills, and executive functions were also affected with a mean frequency approximately of 40%. Intellectual functioning was compromised in 30.3% of patients. Longitudinal studies often reported a decline of neurocognitive functions during the follow-up, indicating a persistent impact of factors like irradiation therapy on cognitive development. To this regard, more cognitive interventions and effective, but less neurotoxic, tumour therapies are needed to preserve or improve neurocognitive functioning and quality of life of this population.

## Author contributions

Supervision and conceptualization, FS. and NV. Writing—original draft preparation, FS, FF. Data curation, FS, FF, BL, GA, DS, GB, and VR. Methodology, EL and NV. Review and editing by all authors. All authors have read and agreed to the published version of the manuscript.
