# Supplementary material for: Frequency and characterization of cognitive impairments in patients diagnosed with paediatric central nervous system tumours: a systematic review
Source: Front Oncol. 2023 May 19;13:1198521. doi: 10.3389/fonc.2023.1198521 (PMC10235613; doi:10.3389/fonc.2023.1198521)
Supplement: Supplementary file 1 [file Table_1.docx]

**Table 1S. External validity of included studies assessed using the MORE tool.**

| **Bibliographic reference** | **External validity** | | | | | | | | | | **Total score** |
| --- | --- | --- | --- | --- | --- | --- | --- | --- | --- | --- | --- |
|  | **1. Sampling of the subjects** | | | **2. Sampling bias** | **3. Estimate bias** | | **4. Exclusion rate** | | **5. Address bias** | **6. Subjects flow** |  |
|  | **General population based** | **Non-general population based method** | **Non-general population based frame** | **Ensure that all members of the reference population had a known chance of selection in the sample** | **Response rate in total sample** | **Response rate in other subgroups** | **Exclusion rate from the analysis** | **Exclusion rate in subgroups** | **Sampling bias addressed in the analysis** | **Subject flow** |  |
| **Prevalence/incidence** | | | | | | | | | | | |
| Aarsen 2004 | NA | 1 | 2 | 1 | 0 | NA | NR | NA | 1 | 0 | **5** |
| Brinkman 2012 | NA | 1 | 2 | NA | 0 | NA | 0 | NA | 1 | 0 | **4** |
| Carpentieri 1993 | NA | 1 | 2 | 1 | 0 | NA | NR | NA | 1 | 0 | **5** |
| Carpentieri 2001 | NA | 1 | 2 | 1 | 0 | NA | NR | NA | 1 | 0 | **5** |
| Cavatorta 2021 | NA | 1 | 2 | 1 | 0 | NA | 0 | NA | 1 | 0 | **5** |
| Child 2021 | NA | 1 | 1 | 1 | 0 | 0 | 0 | 0 | 1 | 0 | **4** |
| Davis 2010 | NA | 1 | 2 | 0 | 0 | 0 | 0 | 0 | 1 | 0 | **4** |
| Docking 2016 | NA | 1 | 2 | 0 | NR | NA | NR | NA | 1 | 2 | **6** |
| Fay-McClymont 2017 | NA | 1 | 2 | NR | 0 | NA | 0 | NA | 0 | 0 | **3** |
| Fen Yang 1997 | NA | 1 | 2 | 2 | NR | NA | 1 | NA | 1 | 1 | **8** |
| Heitzer 2019b | NA | 1 | 2 | NR | 0 | NA | NR | NA | 1 | 0 | **4** |
| Heitzer 2019a | NA | 0 | 0 | 0 | 0 | NA | 0 | NA | 0 | 0 | **0** |
| Holland 2022 | NA | 0 | 0 | 0 | 0 | 0 | 1 | 0 | 0 | 0 | **1** |
| Hoppe-Hirsch 1990 | NA | 0 | 0 | 1 | 1 | NA | NR | NA | 1 | NR | **3** |
| Jacola 2020 | NA | 1 | 2 | 0 | NR | NA | 0 | NA | 0 | 0 | **3** |
| King 2017 | NA | 1 | 1 | 0 | 1 | NA | 0 | NA | 0 | 0 | **3** |
| Kristiansen 2022 | NA | 0 | 0 | 0 | 1 | 0 | 0 | 0 | 1 | 1 | **3** |
| Lacaze 2003 | NA | 1 | 2 | NR | 0 | NA | 0 | NR | 1 | 0 | **3** |
| Lannering 1990 | NA | 1 | 2 | NR | NR | NA | 0 | NA | 1 | 0 | **4** |
| Macedoni-Luksic 2003 | NA | 0 | 0 | 0 | 0 | NA | 2 | NR | 1 | 0 | **3** |
| Maddrey 2005 | NA | 0 | 0 | 0 | 0 | 1 | 2 | NR | 0 | 0 | **3** |
| Packer 1989 | 2 | NA | NA | 2 | 1 | 1 | 1 | 1 | 0 | NR | **8** |
| Palmer 2001 | NA | 0 | 0 | 1 | 0 | NA | 0 | NA | 0 | 1 | **2** |
| Remes 2021 | NA | 1 | 1 | 0 | 0 | NA | 0 | NA | 1 | 0 | **3** |
| Rydén 2022 | NA | 0 | 1 | 0 | 0 | 0 | 0 | 0 | 0 | 1 | **2** |
| Ribi 2005 | NA | 0 | 0 | 1 | 1 | NA | NR | NA | NA | 0 | **2** |
| Rønning 2004 | NA | 1 | 1 | 0 | 1 | NA | NR | NA | 0 | 0 | **3** |
| Rueckriegel 2009 | NA | 2 | 1 | 0 | 0 | NA | 1 | 1 | NR | 0 | **5** |
| Sands 1998 | NA | 1 | 2 | NR | NR | NR | 2 | 2 | NR | 0 | **7** |
| Sands 2010 | 2 | NA | NA | NR | NR | NA | NR | NA | 2 | 0 | **4** |
| Sharkey 2021 | NA | 1 | 1 | 0 | NR | NR | NR | NR | NR | 0 | **2** |
| Söderström 2022 | NA | 1 | 0 | 0 | 0 | NA | 1 | NA | 1 | 1 | **4** |
| Stadskleiv 2020 | NA | 0 | 2 | 1 | NR | NR | 2 | 0 | NR | 0 | **5** |
| Steinlin 2003 | NA | 0 | 2 | NR | 0 | NA | 2 | NA | 1 | 0 | **5** |
| Stensvold 2020 | NA | 0 | 0 | 0 | 0 | NA | 0 | NA | 0 | 0 | **0** |
| Von Hoff 2008 | NA | 1 | 1 | 0 | 0 | NA | 0 | NA | 0 | 1 | **3** |
| Wade 2019 | NA | 1 | 2 | 1 | 2 | NA | 2 | NR | 1 | 0 | **9** |
| Yoo 2016 | NA | 0 | 2 | 1 | NR | NA | NR | NR | 1 | NR | **4** |
| Youn 2022 | NA | 1 | 1 | 0 | 0 | 0 | 0 | 1 | 0 | 1 | **4** |

0= no flaws, 1=Minor flaw, 2=Major flaw, NR=not reported, NA=not applicable

**Table 2S. Internal validity of included studies assessed using the MORE tool.**

| **Bibliographic reference** | **Internal validity** | | | | | | | | | | **Total score** |
| --- | --- | --- | --- | --- | --- | --- | --- | --- | --- | --- | --- |
|  | **1. Source** | **2. Definition of outcome** | | | **3. Outcome measurement** | | **4. Reporting outcomes** | | **5. Outcome in subgroups** | |  |
|  | **Source of measure (incidence/ prevalence)** | **Reference period** | **Severity** | **Frequency of symptoms** | **Validation of outcome measures** | **Reliability of the estimates** | **Reporting of incidence/ prevalence** | **Precision of estimates** | **Prevalence/ incidence in total sample** | **Prevalence/ incidence in subgroups** |  |
| **Prevalence/incidence** | | | | | | | | | | | |
| Aarsen 2004 | 0 | 0 | 0 | 0 | 0 | 0 | 1 | NR | 1 | NA | **2** |
| Brinkman 2012 | 0 | 0 | 0 | 0 | 0 | 0 | 1 | NR | 1 | NA | **2** |
| Carpentieri 1993 | 0 | 0 | 0 | 1 | 0 | 0 | 1 | NR | 1 | NA | **3** |
| Carpentieri 2001 | 0 | 0 | 0 | 1 | 0 | 0 | 1 | NR | 1 | NA | **3** |
| Cavatorta 2021 | 0 | 0 | 0 | 1 | 0 | 0 | 1 | NR | 1 | NA | **3** |
| Child 2021 | 1 | 0 | 0 | 1 | 0 | NR | 1 | NR | NR | 1 | **4** |
| Davis 2010 | 0 | 0 | 0 | 1 | 0 | NR | 1 | NR | 1 | 1 | **4** |
| Docking 2016 | 0 | 0 | 0 | 1 | 0 | 0 | 1 | NR | 1 | NA | **3** |
| Fay-McClymont 2017 | 1 | 0 | 0 | 1 | 0 | NR | 1 | NR | 1 | NA | **4** |
| Fen Yang 1997 | 0 | 0 | 0 | 0 | 0 | NR | NA | NR | NR | NR | **0** |
| Heitzer 2019b | 0 | 0 | 0 | 1 | 0 | NR | 1 | NR | 1 | NA | **3** |
| Heitzer 2019a | 0 | 0 | 1 | 0 | 0 | NA | 0 | 0 | 0 | NA | **1** |
| Holland 2022 | 0 | 0 | 0 | 0 | 0 | 0 | 0 | NR | 0 | 0 | **0** |
| Hoppe-Hirsch 1990 | 2 | 2 | 1 | NR | NR | NR | NR | NR | NR | NR | **5** |
| Jacola 2020 | 1 | 0 | 0 | 0 | 0 | 1 | 1 | NR | 1 | 1 | **5** |
| King 2017 | 0 | 0 | 0 | 0 | 0 | NR | 0 | NR | 1 | NA | **1** |
| Kristiansen 2022 | 0 | 1 | 0 | 0 | 0 | 0 | 0 | 0 | 0 | 1 | **2** |
| Lacaze 2003 | 1 | 0 | 0 | 0 | 0 | NR | 1 | NR | 0 | 0 | **2** |
| Lannering 1990 | 1 | NR | 0 | 0 | NR | 0 | 1 | NR | 1 | NA | **3** |
| Macedoni-Luksic 2003 | 0 | 0 | 0 | 0 | 0 | NR | 1 | NR | 1 | NA | **2** |
| Maddrey 2005 | 0 | 0 | 0 | 0 | 0 | 0 | 1 | NR | 1 | NA | **2** |
| Packer 1989 | 1 | 0 | 0 | NR | 1 | 1 | NR | NR | 0 | NR | **3** |
| Palmer 2001 | 0 | 0 | 0 | 1 | 0 | 1 | 0 | 1 | 0 | NA | **3** |
| Remes 2021 | 0 | 1 | 1 | 0 | 0 | 0 | 0 | 0 | 1 | NA | **3** |
| Rydén 2022 | 1 | 0 | 1 | 0 | 0 | 0 | 0 | 0 | 0 | 1 | **3** |
| Ribi 2005 | NR | 1 | NR | 0 | 0 | 0 | NA | NR | NR | NA | **1** |
| Rønning 2004 | 0 | NR | NR | NR | 0 | 0 | 0 | NR | NR | NA | **0** |
| Rueckriegel 2009 | 0 | NR | NR | NR | 1 | 1 | 2 | 2 | 1 | NR | **7** |
| Sands 1998 | 1 | 0 | 0 | 0 | 2 | 2 | 1 | 0 | NR | NA | **6** |
| Sands 2010 | 0 | 0 | 0 | 0 | 0 | 0 | 0 | 0 | 0 | NA | **0** |
| Sharkey 2021 | 0 | 0 | 0 | 0 | 0 | 0 | 0 | 0 | 0 | NA | **0** |
| Söderström 2022 | 0 | 0 | 0 | 1 | 0 | 1 | 2 | 0 | 0 | NA | **4** |
| Stadskleiv 2020 | 1 | 0 | 0 | 0 | 0 | NR | 1 | 0 | 1 | 1 | **4** |
| Steinlin 2003 | 0 | 0 | 0 | 0 | 0 | NR | 1 | 0 | 1 | NA | **2** |
| Stensvold 2020 | 0 | 0 | 0 | 0 | 0 | NR | 1 | 0 | 1 | 1 | **3** |
| Von Hoff 2008 | 0 | 1 | 1 | 0 | 0 | 0 | 0 | 0 | 1 | NA | **3** |
| Wade 2019 | 0 | 0 | 0 | 0 | 0 | NR | NA | NR | NR | NA | **0** |
| Yoo 2016 | 0 | 0 | 0 | 0 | 0 | NR | 0 | NR | NR | NA | **0** |
| Youn 2022 | 0 | 0 | 0 | 0 | 0 | NR | 0 | NR | NR | 1 | **1** |

0= no flaws, 1=Minor flaw, 2=Major flaw, NR=not reported, NA=not applicable
