# Supplementary material for: Frequency and characterization of cognitive impairments in patients diagnosed with paediatric central nervous system tumours: a systematic review
Source: Front Oncol. 2023 May 19;13:1198521. doi: 10.3389/fonc.2023.1198521 (PMC10235613; doi:10.3389/fonc.2023.1198521)
Supplement: Supplementary file 2 [file Table_3.docx]

| **Table 3S** |
| --- |
| **References of Supplementary Material** |
| - Aarsen FK, Van Dongen HR, Paquier PF, Van Mourik M, Catsman-Berrevoets CE. Long-term sequelae in children after cerebellar astrocytoma surgery. Neurology (2004) 62(8):1311–6. doi: 10.1212/01.wnl.0000120549.77188.36; |
| - Brinkman TM, Reddick WE, Luxton J, Glass JO, Sabin ND, Srivastava DK, et al. Cerebral white matter integrity and executive function in adult survivors of childhood medulloblastoma. Neuro Oncol (2012) 14 Suppl 4(Suppl 4):iv25–36. doi: 10.1093/ neuonc/nos214; |
| - Carpentieri SC, Mulhern RK. Patterns of memory dysfunction among children surviving temporal lobe tumors. Arch Clin Neuropsychol. (1993) 8(4):345–57; |
| - Carpentieri SC, Waber DP, Scott RM, Goumnerova LC, Kieran MW, Cohen LE, et al. Memory deficits among children with craniopharyngiomas. Neurosurgery (2001) 49(5):1053–7; discussion 1057-8. doi: 10.1097/00006123-200111000-00005; |
| - Cavatorta C, Meroni S, Montin E, Oprandi MC, Pecori E, Lecchi M, et al. Retrospective study of late radiation-induced damages after focal radiotherapy for childhood brain tumors. PloS One (2021) 16(2):e0247748. doi: 10.1371/ journal.pone.0247748; |
| - Child AE, Warren EA, Grosshans DR, Paulino AC, Okcu MF, Ris MD, et al. Long-term cognitive and academic outcomes among pediatric brain tumor survivors treated with proton versus photon radiotherapy. Pediatr Blood Cancer. (2021) 68(9): e29125. doi: 10.1002/pbc.29125; |
| - Davis EE, Pitchford NJ, Jaspan T, McArthur D, Walker D. Development of cognitive and motor function following cerebellar tumour injury sustained in early childhood. Cortex (2010) 46(7):919–32. doi: 10.1016/j.cortex.2009.10.001; |
| - Docking K, Munro N, Marshall T, Togher L. Narrative skills of children treated for brain tumours: the impact of tumour and treatment related variables on microstructure and macrostructure. Brain Inj. (2016) 30(8):1005–18. doi: 10.3109/ 02699052.2016.1147602; |
| - Fay-McClymont TB, Ploetz DM, Mabbott D, Walsh K, Smith A, Chi SN, et al. Long-term neuropsychological follow-up of young children with medulloblastoma treated with sequential high-dose chemotherapy and irradiation sparing approach. J Neurooncol. (2017) 133(1):119–28. doi: 10.1007/s11060-017-2409-9; |
| - Heitzer AM, Ashford JM, Hastings C, Liu APY, Wu S, Bass JK, et al. Neuropsychological outcomes of patients with low-grade glioma diagnosed during the first year of life. J Neurooncol. (2019) 141(2):413–20. doi: 10.1007/s11060-018- 03048-0; |
| - Heitzer AM, Raghubar K, Ris MD, Minard CG, Gragert MN, Stancel HH, et al. Neuropsychological functioning following surgery for pediatric low-grade glioma: a prospective longitudinal study. J Neurosurg Pediatr (2019) 6:1–9. doi: 10.3171/ 2019.9.PEDS19357; |
| - Holland AA, Shamji JF, Clem MA, Perez R, Palka JM, Stavinoha PL. Parent Q18 ratings of executive functioning in pediatric survivors of medulloblastoma and pilocytic astrocytoma. Appl Neuropsychol Child. (2022), Sep 16:1–10. doi: 10.1080/ 21622965.2022.2123707; |
| - Hoppe-Hirsch E, Renier D, Lellouch-Tubiana A, Sainte-Rose C, Pierre-Kahn A, Hirsch JF. Medulloblastoma in childhood: progressive intellectual deterioration. Childs Nerv Syst (1990) 6(2):60–5. doi: 10.1007/BF00307922; |
| - Jacola LM, Anghelescu DL, Hall L, Russell K, Zhang H, Wang F, et al. Anesthesia exposure during therapy predicts neurocognitive outcomes in survivors of childhood medulloblastoma. J Pediatr (2020) 223:141–147.e4. doi: 10.1016/j.jpeds.2020.04.039; |
| - King TZ, Ailion AS, Fox ME, Hufstetler SM. Neurodevelopmental model of long-term outcomes of adult survivors of childhood brain tumors. Child Neuropsychol. (2017) 25(1):1–21. doi: 10.1080/09297049.2017.1380178; |
| - Kristiansen I, Eklund C, Strinnholm M, Strömberg B, Törnhage M, Frisk P. Cognitive, language, and school performance in children and young adults treated for low-grade astrocytoma in the posterior fossa in childhood. Cancer Rep (Hoboken). (2022) 5(3):e1494. doi: 10.1002/cnr2.1494; |
| - Lacaze E, Kieffer V, Streri A, Lorenzi C, Gentaz E, Habrand JL, et al. Neuropsychological outcome in children with optic pathway tumours when first-line treatment is chemotherapy. Br J Cancer. (2003) 89(11):2038–44. doi: 10.1038/ sj.bjc.6601410; |
| - Lannering B, Marky I, Lundberg A, Olsson E. Long-term sequelae after pediatric brain tumors: their effect on disability and quality of life. Med Pediatr Oncol (1990) 18 (4):304–10. doi: 10.1002/mpo.2950180410; |
| - Macedoni-Luksic M, Jereb B, Todorovski L. Long-term sequelae in children treated for brain tumors: impairments, disability, and handicap. Pediatr Hematol Oncol (2003) 20(2):89–101. doi: 10.1080/0880010390158595; |
| - Maddrey AM, Bergeron JA, Lombardo ER, McDonald NK, Mulne AF, Barenberg PD, et al. Neuropsychological performance and quality of life of 10 year survivors of childhood medulloblastoma. J Neurooncol. (2005) 72(3):245–53. doi: 10.1007/s11060-004-3009-z; |
| - Packer RJ, Sutton LN, Atkins TE, Radcliffe J, Bunin GR, D’Angio G, et al. A prospective study of cognitive function in children receiving whole-brain radiotherapy and chemotherapy: 2-year results. J Neurosurg (1989) 70(5):707–13. doi: 10.3171/ jns.1989.70.5.0707; |
| - Palmer SL, Goloubeva O, Reddick WE, Glass JO, Gajjar A, Kun L, et al. Patterns of intellectual development among survivors of pediatric medulloblastoma: a longitudinal analysis. J Clin Oncol (2001) 19(8):2302–8. doi: 10.1200/ JCO.2001.19.8.2302; |
| - Remes TM, Hovén E, Ritari N, Pohjasniemi H, Puosi R, Arikoski PM, et al. Neurocognitive impairment, employment, and social status in radiotherapy-treated adult survivors of childhood brain tumors. Neurooncol Pract (2021) 8(3):266–77. doi: 10.1093/nop/npab004; |
| - Ribi K, Relly C, Landolt MA, Alber FD, Boltshauser E, Grotzer MA. Outcome of medulloblastoma in children: long-term complications and quality of life. Neuropediatrics (2005) 36(6):357–65. doi: 10.1055/s-2005-872880; |
| - Rønning C, Sundet K, Due-Tønnessen B, Lundar T, Helseth E. Persistent cognitive dysfunction secondary to cerebellar injury in patients treated for posterior fossa tumors in childhood. Pediatr Neurosurg (2005) 41(1):15–21. doi: 10.1159/ 000084860; |
| - Rueckriegel SM, Blankenburg F, Henze G, Baqué H, Driever PH. Loss of fine motor function correlates with ataxia and decline of cognition in cerebellar tumor survivors. Pediatr Blood Cancer. (2009) 53(3):424–31. doi: 10.1002/pbc.22104; |
| - Rydén I, Fernström E, Lannering B, Kalm M, Blomstrand M, Hellström P, et al. Neuropsychological functioning in childhood cancer survivors following cranial radiotherapy - results from a long-term follow-up clinic. Neurocase (2022) 28 (2):163–72. doi:10.1080/13554794.2022.2049825; |
| - Sands SA, Oberg JA, Gardner SL, Whiteley JA, Glade-Bender JL, Finlay JL. Neuropsychological functioning of children treated with intensive chemotherapy followed by myeloablative consolidation chemotherapy and autologous hematopoietic cell rescue for newly diagnosed CNS tumors: an analysis of the head start II survivors. Pediatr Blood Cancer. (2010) 54(3):429–36. doi: 10.1002/pbc.22318; |
| - Sands SA, van Gorp WG, Finlay JL. Pilot neuropsychological findings from a treatment regimen consisting of intensive chemotherapy and bone marrow rescue for young children with newly diagnosed malignant brain tumors. Childs Nerv Syst (1998) 14(10):587–9. doi: 10.1007/s003810050277; |
| - Sharkey CM, Mullins LL, Clawson AH, Gioia A, Hawkins MAW, Chaney JM, et al. Assessing neuropsychological phenotypes of pediatric brain tumor survivors. Psychooncology (2021) 30(8):1366–74. doi: 10.1002/pon.5692; |
| - Söderström H, Brocki K, Kleberg JL, Martinsson U, Ljungman G. Neurocognitive functions before and after radiotherapy in pediatric brain tumor survivors. Pediatr Neurol (2022) 133:21–9. doi: 10.1016/j.pediatrneurol.2022.05.006; |
| - Stadskleiv K, Stensvold E, Stokka K, Bechensteen AG, Brandal P. Neuropsychological functioning in survivors of childhood medulloblastoma/CNSPNET: the role of secondary medical complications. Clin Neuropsychol. (2020) 36 (3):600–25. doi: 10.1080/13854046.2020.1794045; |
| - Steinlin M, Imfeld S, Zulauf P, Boltshauser E, Lövblad KO, Ridolfi Lüthy A, et al. Neuropsychological long-term sequelae after posterior fossa tumour resection during childhood. Brain (2003) 126(Pt 9):1998–2008. doi: 10.1093/brain/awg195; |
| - Stensvold E, Stadskleiv K, Myklebust TÅ, Wesenberg F, Helseth E, Bechensteen AG, et al. Unmet rehabilitation needs in 86% of Norwegian paediatric embryonal brain tumour survivors. Acta Paediatr (2020) 109(9):1875–86. doi: 10.1111/apa.15188; |
| - von Hoff K, Kieffer V, Habrand JL, Kalifa C, Dellatolas G, Grill J. Impairment of intellectual functions after surgery and posterior fossa irradiation in children with ependymoma is related to age and neurologic complications. BMC Cancer. (2008) 8:15. doi: 10.1186/1471-2407-8-15; |
| - Wade SL, Narad ME, Moscato EL, LeBlond EI, King JA, Raj SP, et al. A survivor’s journey: preliminary efficacy of an online problem-solving therapy for survivors of pediatric brain tumor. Pediatr Blood Cancer. (2020) 67(2):e28043. doi: 10.1002/pbc.28043; |
| - Yang TF, Wong TT, Cheng LY, Chang TK, Hsu TC, Chen SJ, et al. Neuropsychological sequelae after treatment for medulloblastoma in childhood–the Taiwan experience. Childs Nerv Syst (1997) 13(2):77–80; discussion 81. doi: 10.1007/ s003810050046; |
| - Yoo HJ, Kim H, Park HJ, Kim DS, Ra YS, Shin HY. Neurocognitive function and health-related quality of life in pediatric Korean survivors of medulloblastoma. J Korean Med Sci (2016) 31(11):1726–34. doi: 10.3346/jkms.2016.31.11.1726; |
| - Youn SH, Ha B, Lee EH, Park B, Yang SE, Yu ES, et al. Neurocognitive and psychological functioning of pediatric brain tumor patients undergoing proton beam therapy for three different tumor types. Pediatr Blood Cancer. (2022) 69(3):e29430. doi: 10.1002/pbc.29430. |
